# When Feeling Skillful Impairs Coordination in a Lottery Selection Task

**DOI:** 10.1371/journal.pone.0065092

**Published:** 2013-06-14

**Authors:** Anna Dorfman, Yoella Bereby-Meyer, Simone Moran

**Affiliations:** 1 Department of Psychology, Ben Gurion University of the Negev, Beer Sheva, Israel; 2 Gilford Glazer Faculty of Business and Management, Ben Gurion University of the Negev, Beer Sheva, Israel; Brain and Spine Institute (ICM), France

## Abstract

Choosing a major field of study to secure a good job after graduation is a tacit coordination problem that requires considering others’ choices. We examine how feeling skillful, either induced (Experiment 1) or measured (Experiment 2), affects coordination in this type of task. In both experiments participants chose between two lotteries, one offering a larger prize than the other. Participants’ entry into the chosen lottery was either related or unrelated to their skill, with the final prize allocated randomly to one of the entrants in each lottery. Importantly, across conditions skill was irrelevant to choosing *between* lotteries. Notwithstanding, when skill was related to determining lottery entrants, participants who felt highly skillful chose the high prize lottery excessively. Results further suggest that this stems from high confidence in self skill, rather than incorrect expectations regarding others.

## Introduction

The outcomes of choices and actions in social environments are often interdependent, and involve others’ actions. For example, when driving to work, the consequence of taking the route with eighty miles of highway rather than the alternative route with ninety miles of highway depends on the simultaneous choices of other drivers. Similarly, when choosing a major field of study, the consequence of choosing a major that leads to a higher paying profession (e.g., software engineering) over a lower paying one (e.g., mechanical engineering) depends on other students’ decisions. Although software engineering could seem like the more promising alternative because this profession pays better, if too many others choose this major the market might be flooded with software engineers, in which case the seemingly less attractive mechanical engineering would be a better alternative. This problem, as well as other common everyday choices (e.g. the decision to use public vs. private transportation during rush hour or whether to buy more lottery tickets when the jackpot increases) requires tacit coordination.

In tacit coordination choice problems, decision makers can choose only one of several independent and mutually exclusive alternatives, and the outcome of their choice depends on the number of decision makers who choose the same alternative. Hence, the optimal behavior is defined not only in terms of the utility of each outcome, but also in terms of the estimated number of participants who choose each alternative.

The above tacit coordination choice problems can be modeled as a lottery selection task (i.e. a non-cooperative game with *N* players). In a lottery selection task individuals have to choose simultaneously and without communication which of two (or more) lotteries with varying jackpots to enter – the higher the number of participants who choose each lottery, the lower the expected value of the lottery for any given participant. Consequently, the challenge of successful coordination is in accurately predicting others’ preferences and decisions and taking them into consideration when making the decision.

Previous tacit coordination studies with a lottery selection task typically revealed coordination success. Rapoport, King-Chung Lo, and Zwick (2002) asked groups of 18 participants to choose repeatedly one of three lotteries with varying prizes (e.g. $14, $12, $10). For each trial, one winner was randomly selected from each of the three lotteries. Therefore, the probability of winning a prize in the lottery decreased as a function of the number of players choosing it. This study revealed a strong pattern of tacit coordination achieved through repeated trials [1]. Recently, Bereby-Meyer, Moran, Grosskopf, and Chugh (in press) presented 200 participants with a one-shot lottery selection task without communication or information about the equilibrium solution. The authors determined the winning number of the lottery (i.e., a number between 0 and 9) randomly. In each of these lotteries either all guessers of the winning number shared the prize equally, or a randomly-selected one received it. Surprisingly, the authors observed almost perfect coordination across treatments. Namely, the distribution of participants’ lottery choices did not significantly differ from the theoretical equilibrium solution [2].

Successful coordination was also reported in a different coordination task, known as the market entry game. In a typical market entry game, *N* players make repeated individual decisions of whether to enter an idealized competitive market with a known capacity *C*. If the number of players that enter the market exceeds market capacity, entry may lead to negative payoff. Surprisingly, the number of players who chose to enter the market in several laboratory experiments was close to the number that theory predicts (i.e. around *C* entrants), even though all players made their choices simultaneously and without communication – a phenomenon referred to as “magic” [3], [4], [5], [6].

Notably, however, people in real world economic settings often do not coordinate well. Market entry decisions do not seem to reflect perfect coordination, and most new businesses fail within a few years [7], [8], [9]. The discrepancy between the coordination that is observed in most market entry games in the lab and the failure to coordinate that is observed in reality may be due to psychological factors, such as overconfidence, which were irrelevant to the coordination tasks that had previously been studied in the lab [10], [11]. Consistent with this notion, potential entrants in another study, based their decision to enter primarily on evaluations of their own competence (or incompetence) and paid relatively little attention to the strength of the competition, demonstrating egocentric biases in market entry decisions [12].

In the current study we explore how feeling skillful affects tacit coordination in a lottery selection task that does or does not involve skill. Based on findings from market entry games and from a recent study [2], we hypothesize that when skill is unrelated to task outcomes, as was the case in previous lottery selection task studies, coordination will be achieved. However, when skill is related to task outcomes, we expect participants who feel highly skillful to focus mainly on their self-perceived skill and to over-choose the high-prize lottery.

It is important to note that while the market entry game and the lottery selection task both require coordination for accomplishing optimal performance, they are significantly different in other respects. One major difference is the way the options from which one must choose are designed. In the market entry game the option to enter the market requires an active decision, while the option to stay out of the market is the default. Thus, in the market entry game the decision to enter the market may be confounded by the tendency to choose the active over the passive (i.e. default) alternative. Previous findings suggest that feeling powerful increases the tendency to choose the active alternative [13]. Since feelings of confidence (i.e. high skillfulness) and power are likely to be related, it is possible that feeling skillful does not affect entry directly, but rather the tendency to choose the active alternative.

The lottery selection task that we use in the current study simulates situations in real life where there is no default option, for example choosing to major in computer science or mechanical engineering. In this type of task the two alternatives require equally active choices, i.e. there is no default option. Thus the lottery selection task controls for an alternative action versus inaction account. Moreover, the market entry game has a business context, which may boost effects of overconfidence on market entry decisions. The lottery selection task is a more neutral and context-free paradigm.

### Overview of Study

We conducted two experiments using the paradigm of a one-shot lottery selection task [2]. In both experiments, the lottery selection task includes two stages. First, participants are informed that *N* players, including themselves, are to choose whether to participate in a high-prize or a low-prize lottery. Second, after the choice is made, actual entry into the chosen lottery is determined by performance on a given task. Importantly, the instructions stress that the task is the same for both lotteries. In each lottery, one winner is randomly selected out of all entrants to receive the prize.

We define the expected value of each lottery (L_i_) as follows (for the derivation of Equation (1), see [2]).
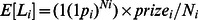
(1)


*N_i_ = Number of entrants in lottery L*_i_

*p*_i_ *= Probability of entering lottery L*_i_

*Prize _i_ = Amount of the prize in lottery L*_i_

In Experiment 1 we induced a feeling of being skilled by giving all participants extreme positive feedback regarding an unfamiliar (bogus) skill. In Experiment 2 we measured participants’ perceptions regarding their pre-existing knowledge in soccer. Across both experiments we manipulated the relevance of skill to task outcome; namely, participants’ skill was either related (“self” condition) or unrelated (“computer” condition) to the outcome of the task that determined entry into the chosen lottery. Our computer condition was compatible with previously studied coordination games in which outcomes were determined randomly [2], [3], [4], [5], [6]. This manipulation allowed us to explore how feeling skillful may affect coordination when outcomes are related versus unrelated to skill.

Importantly, although in some conditions the task involved skill, we designed our experiments such that skill was irrelevant to the choice between lotteries. Specifically, in Experiment 1 all participants were similarly skillful and this was common knowledge to all, and in Experiment 2 participants were recruited based on their high reported knowledge in soccer and the task we used required predicting the outcome of soccer games, where knowledge plays a minor role [14].

The current study extends previous studies, and particularly the work demonstrating effects of over confidence on deviation from equilibrium in market entry games [10], with respect to a number of key aspects. First, as mentioned above, the lottery selection task we use includes no default option- i.e., there is no status quo. This enables to control for a tendency to choose the more over the less active alternative, which may account for previous excessive market entry findings [10], [15]. Related to this point, in our lottery selection task both options involve uncertainty regarding the prize to be won, whereas in the typical market entry game the payoff for not entering (i.e., the default option) is fixed. Moreover, the lottery selection task in the current study is a one-shot task, thus eliminating the possibility of learning to coordinate through repeated trials.

## Experiment 1

In the first experiment we examined the ability of participants who feel highly skilled to coordinate in a lottery selection task. We induced a feeling of skillfulness by providing participants with a task and feedback in a domain in which they did not have initial knowledge or perceptions regarding their skills. We then manipulated whether entering the lottery depended on skill or not by designing two conditions: a self condition in which participants’ performance affected their entry, and a computer condition in which entry was determined by a computer program.

### Methods

The research has been approved by the authors’ departmental institutional review board.

#### Participants

Ninety undergraduate students participated as part of a longer session consisting of a number of unrelated experiments, and were paid for their participation.

Importantly, since this experiment was for real money, to conform to the precise number of participants that was mentioned in the instructions and that also served as the basis for computing the equilibrium, we continued to run the experiment until we reached the target of forty participants in each condition who passed our task understanding selection criteria.

#### Procedure

We ran the experiment in group sessions of three to six participants who sat separately and performed the task individually on a computer. We assigned participants randomly to either the self or the computer condition in a between-subjects design.

First, we induced a feeling of being highly skilled by presenting participants with a novel and bogus task allegedly measuring “Intuitive Quantitative Perception” (IQP). We told participants that people differ in their IQP skill and that some have a higher IQP skill than others. Importantly, we described this skill as intuitive and instructed participants to respond according to their “gut feeling”, even if they were not certain of the correct response in each trial. Participants performed 100 trials of the IQP task. In each trial a target composed of a varying number of dots (10–99) was presented briefly (50 milliseconds). After the target disappeared from the screen, we asked participants to estimate how many dots composed the target and to select the appropriate interval in which the number fell (e.g. if she/he estimated the target as composed of 55 dots, she/he was to press “5,” which indicated an interval between 50 and 59). Unrelated to participants’ actual performance, on 90 percent of the trials they received positive feedback. For the task and feedback to appear real, we presented negative feedback on 10 percent of the trials.

Upon completing the task, we informed the participants that they had scored high on the task and that they possessed superior IQP skills. Subsequently, we informed them that a group of 40 participants (including themselves) who had received similar high IQP scores would have the opportunity to participate in one of two lotteries, in which they could possibly earn cash prizes of 100 Israeli Shekels (hereafter IS) in the low-prize lottery versus 200 IS in the high-prize lottery (see Text_S1).

Before choosing which of the two lotteries they wanted to participate in, we informed participants in the self condition that their participation in the lottery they chose would depend on their performance in an additional trial of the IQP task. We specifically told them that only participants who responded correctly in this additional trial would enter the lottery.

We informed the participants in the computer condition that their actual participation in the lottery they chose would depend on their outcome in an additional trial of the IQP task. We explained that for each participant a computer program would evaluate the number of dots presented, and that the probability of this program correctly evaluating is close to 99 percent. We specifically told them that only those participants for whom the program selected a number that correctly matched the number of dots presented would enter the lottery. In both conditions we explained that if more than one participant entered the lottery, we would randomly select one winner to receive the prize.

After reading the instructions and choosing a lottery, participants reported their expectations regarding others’ choices (i.e., how many out of the 40 participants chose each lottery). We then asked participants to rate on a scale from 1 (very low skill) to 9 (very high skill) their own IQP skill, the IQP of another participant (out of the 40 high-score participants), and the probability that they and another participant would perform the task correctly (i.e., enter into the lottery).

To confirm understanding of the task, we asked the participants how the final task was performed (i.e., by a computer program or by the participants themselves). This question served to ensure that participants in the computer condition understood that their skills were unrelated to the task outcome.

Finally, participants in the self condition performed the task (in the computer condition the computer program assigned the correct numbers for all participants) and we thanked them all for their participation. We conducted the lotteries and notified all participants via e-mail regarding the winners (one from those that chose the high-prize lottery and one from those that chose the low-prize lottery, for each condition), who received their cash prize.

#### Nash equilibrium

The Nash equilibrium solution (i.e. defined as a solution in which the expected values of both lotteries were equal) was similar across both conditions. For the self condition, we assumed that since participants were told that all participants (including themselves) performed equally high, they should have expected that all participants have an equal probability to enter the lottery. Consequently, the equilibrium solution based on equation (1) was 67 percent of participants choosing the high-prize lottery and 33 percent choosing the low-prize lottery. For the computer condition, the probability that the computer program would correctly evaluate the number of dots was 0.99, and the equilibrium was almost the same (68 percent choosing the high-prize lottery).

### Results and Discussion

We excluded five participants because they did not understand the instructions in the computer condition. We excluded five additional participants (three in the self condition and two in the computer condition) because their reported expectations about the number of other participants who chose each lottery did not add up to 40 (the total number of participants in each condition) and therefore they could not be coded properly. Overall, we analyzed 80 participant responses (40 in each experimental condition) and report the results below.

#### Distribution of choices

Table 1 summarizes the distribution of participants across lotteries for the self and computer conditions. In line with our predictions, the observed distribution of choices in the computer condition did not differ from the distribution expected in equilibrium, (χ2(1) = 0.005, p = 0.95). Twenty-seven out of forty participants in this condition (67.5%) chose the high-prize lottery. More importantly, in the self condition, in line with our prediction, the observed distribution of choices differed from the distribution expected in equilibrium, (χ2(1) = 4.35, p<0.05). Thirty three out of forty participants (82.5%) chose the high-prize lottery.

**Table 1:** Choice of high vs. low lottery by condition (Experiment 1).

Condition	Low Prize Lottery	High Prize Lottery
Self	17. 5% (7)	82.5% (33)
Computer	32.5% (13)	67.5% (27)
*Equilibrium*	33%	67%

Note: The actual number of participants in each cell is presented in parentheses.

Two possible explanations may be offered for the deviation from equilibrium we observe in the skill condition: (1) inaccurate expectations regarding others’ choices, or (2) accurate expectations that were not taken into consideration. According to the first explanation, participants’ expectations of the number of others that chose the high-prize lottery were inaccurate (i.e., participants underestimated the number of others choosing the high-prize lottery). In that case, participants may have chosen correctly, given their (inaccurate) expectations.

Alternatively, the second explanation states that participants’ expectations of others’ choices were accurate (i.e. they expected most participants to choose the high-prize lottery). However, these expectations were insufficiently taken into consideration, possibly due to the participants being overconfident about their own skill relative to others’ skill. This overconfidence may have motivated them to choose the high-prize lottery, ignoring their (accurate) expectations that most others will choose it as well.

We next examined these two possible explanations. We started by computing each participant’s expected value from choosing each lottery, given her/his expectations regarding other participants’ choices. Next, given their actual choice (high-prize lottery/low-prize lottery), we computed their foregone payoff, namely their expected gain or loss over their expected gain or loss if they would have chosen the alternative lottery. For example, a participant who in the self condition expected that 20 participants would choose the high-prize lottery has an expected value of 10 for choosing the high prize lottery based on Equation 1, while the expected value for choosing the low prize lottery is 5. Assuming that this participant chose the low prize lottery, given the expected values of the two lotteries, this participant forewent 4.52 NIS. This foregone profit is the difference between the expected value from choosing the low prize lottery (5) and the expected value from choosing the high-prize lottery, based on 20+1 participants who chose it (9.52).

The mean foregone profit for the self condition was (−32.27), Sd = 45.67 and the mean for the computer condition was (−22.44), Sd = 43.82. Thus in the self condition, given participants’ choices and expectations, on average their expected loss was 32.27 IS, compared to only 22.44 IS in the computer condition. This analysis suggests that the reason for the excessive choice of the high-prize lottery is not inaccurate expectations of others’ that are taken into consideration correctly. Rather, this excessive choice may be due to participants’ overconfidence in their self-skill relative to others, and consequent neglect or insufficient consideration of others’ choices.

Indeed, in the self condition participants assessed their own skill as significantly higher (M = 7.20, Sd = 0.97) than another participant’s skill (M = 6.47, Sd = 1.26), (t(39) = 3.47, p<0.01), and accordingly assessed their own chance to enter the lottery as higher (M = 6.57, Sd = 1.17) than another participant’s chance (M = 6.27, Sd = 1.30), t(39) = 2.02, p = 0.05. It is important to note that this overconfidence does not seem to be a result of unawareness of the fact that other participants were also skillful. Participants did estimate another’s skill as high and well above intermediate (mean = 6.5, on a 9 point scale), even though lower than their own estimated skill. In the computer condition participants also assessed their own skill (M = 7.60, Sd = 1.01) as significantly higher than another participant’s skill (M = 6.55, Sd = 1.20), (t(39) = 5.27, p<0.001). However, interestingly, they did not assess their own chance of entering the lottery as higher than another participant’s chance. In fact, when skill was unrelated to task outcome, participants assessed their own chance of entering the lottery to be even lower (M = 6, Sd = 2.42) than another participant’s chance (M = 6.75, Sd = 2.11), t(39) = −2.62, p<0.05. We further elaborate on this finding in the general discussion.

## Experiment 2

Experiment 2 aimed to generalize the main finding of Experiment 1, namely that feeling skillful impairs coordination, by replicating it in a task that was not novel, and measuring (rather than inducing) participants’ existing subjective knowledge. Participants were given a hypothetical task of predicting soccer games’ outcomes. Similar to Experiment 1, we used the lottery selection task and manipulated whether entering the lottery did or did not depend on participants’ prediction (i.e., whether skill was or was not related to task outcome). We chose the task of predicting soccer games’ outcomes since this is a task for which skill has a minor impact on outcomes [14]. Specifically, experts (i.e., sport journalists, soccer fans, and soccer coaches) were not better than non-experts in predicting the outcome of the first round of World Cup 2002. Furthermore, experts overestimated their performance and tended to be overconfident.

We expected to find a correlation between estimated knowledge and choosing the high lottery only when entering the lottery depended on one’s prediction, and not when it was determined by a computer program.

### Methods

#### Participants

One hundred and one undergraduates volunteered to participate in this study, on the basis of having knowledge in soccer.

#### Procedure

We randomly assigned participants to either a self or computer condition and asked them to imagine that the experiment involved 200 participants (including themselves) who have to choose one out of two lotteries: 250 IS versus 500 IS (see Text_S2).

Before choosing, we told participants in the self condition that entry into the chosen lottery depends on correctly predicting the results of three soccer games. In the computer condition we told participants that for each participant, a computer program would predict the results of the three soccer games (i.e. team 1 wins; team 2 wins; tie) and that they could not affect this process. In both conditions, only participants with three correct predictions were eligible to enter the lottery. As in Experiment 1, across both conditions, the scenario stated that if more than one participant entered a lottery, one of would be randomly selected as the prize winner.

After reading the scenario, participants chose one of the two lotteries and reported their expectations regarding others’ choices (i.e. how many out of 200 participants would be likely to choose each lottery). We also asked them to rate their own soccer knowledge on a scale ranging from 0 (no knowledge at all) to 10 (perfect knowledge).

#### Nash equilibrium

We calculated the Nash equilibrium solution for each condition. The probability of a participant with perfect knowledge to enter lottery L is 1, and the probability of entering lottery L by guessing all three results correctly (by a participant with no knowledge or by a random device) is 0.04. Based on equation (1), for 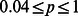, the equilibrium is 67%–68% of participants choosing the high-prize lottery and 32%–33% choosing the low-prize lottery. Thus the equilibrium in this case is relatively insensitive to the probability of guessing the results of the three soccer games correctly.

### Results and Discussion

We excluded two participants from the analysis because they failed to report their expectations about the choices of others. Thus we analyzed 99 participant responses (51 in the self condition and 48 in the computer condition) and present the results below.

#### Self-skill estimates and choice of lottery

We started by analyzing the effect of estimated skill on choice of lottery in the different conditions. Because participants were recruited based on being knowledgeable in soccer, the distribution of skill perception was negatively skewed. Thus we coded their skill based on the median (Md = 8) as being either relatively high (above the median), moderate (median), or low (below the median). We conducted a logistic regression analysis predicting choice of lottery as the dependent variable (High prize = 1, Low prize = 0), from condition (Self = 1, Computer = 0), self-knowledge estimate (coded as relatively high, moderate, or low) represented by two dummy variables (High = 1, Moderate = 1) with the low self-knowledge group as the reference group, and condition by self-knowledge estimate interaction, as independent variables.

The logistic regression revealed a significant condition by High self-knowledge estimate interaction (b = 2.23, p = 0.05). No other effects were significant.

Next, in each condition we examined the distribution of choice as a function of participants’ estimated skill. Table 2 summarizes the results.

**Table 2:** Choice of high vs. low lottery in the self vs. computer conditions as a function of self-knowledge estimates (High, Moderate, Low).

	Computer condition		Self condition	
Estimated Knowledge	Low Prize Lottery	High Prize Lottery	Low Prize Lottery	High Prize Lottery
Low	19.2% (5)	80.8% (21)	40.9% (9)	59.1% (13)
Moderate	23.1% (3)	76.9% (10)	28.6% (4)	71.4% (10)
High	44.4% (4)	55.6% (5)	20% (3)	80% (12)
Overall	25% (12)	75% (36)	31.4% (16)	68.6% (35)
*Equilibrium*	32%	68%	33%	67%

Note: The actual number of participants in each cell is presented in parentheses.

Table 2 demonstrates, as expected, that in the self condition, high estimated self-knowledge resulted in a greater tendency to choose the high prize lottery (80%), compared to low estimated self-knowledge (59.1%). Interestingly, a reversed pattern was apparent in the computer condition, where only 55.6% of the participants with high estimated self-knowledge chose the high prize lottery and 80.8% of the relatively low estimated self-knowledge chose the high prize lottery. We elaborate on this observation in the discussion.

It is worth noting that the distribution of participants for the different lotteries across estimated skill was close to equilibrium regardless of the relevance of skill to task outcome. We observed coordination in the computer condition (36 out of 48 participants [75%] chose the high-prize lottery, not significantly different from the equilibrium prediction, p = 0.3) as well as in the self condition (35 out of 51 participants [68.6%] chose the high-prize lottery, not significantly different from the equilibrium prediction, p = 0.8).

## Discussion

We explored decision makers’ tendencies to coordinate when feeling skillful and facing a lottery selection task that does versus does not involve skill. Previous studies have shown that participants often succeed in achieving coordination in such tacit coordination tasks [1], [2], [3], [4], [5], [6].We suggest that this is the case as long as skill is unrelated to the task.

When outcomes are contingent on skill, coordination is susceptible to participants’ feelings of skillfulness. Experiment 1 shows that participants who feel highly skilled fail to achieve tacit coordination due to their tendency to over-choose the high-prize lottery, despite knowing that the other participants are highly skilled, too. This excessive choice of the high-prize lottery does not result from accurate consideration of an inaccurate estimation of the number of participants who chose the high lottery, since the accuracy of participants’ choices based on their reported expectations regarding choices of others is low. Rather, the excessive choice of the high-prize lottery seems to reflect participants’ insufficient consideration of others’ abilities and choices. Our results further suggest that this may be due to overconfidence in one’s own skill compared to others, and a corresponding optimistic evaluation of the chances to perform well compared to others. This is in line with the observed difficulty in reaching coordination in market entry game studies in which participants were overconfident in their relative skill [10], [11], [12], [16].

Interestingly, overconfidence in skill does not always lead to a more general overconfidence or optimistic bias. In the computer condition, participants rated their own skill as higher than the average participant’s skill (i.e., they too demonstrate overconfidence in self-skill). However, they unexpectedly rated their own chance to enter the lottery as lower than the average participant’s chance. It is possible that when highly-skilled individuals cannot use their skills to improve their outcome, being skillful leads to pessimism rather than optimism. Further research is needed to shed light on this compelling notion.

Experiment 2 further shows that participants’ subjective knowledge estimates in soccer predict their tendency to choose the high-prize lottery. Specifically, in the self condition, it is those who feel more knowledgeable relative to others who excessively choose the high-prize lottery and consequently fail to reach coordination. Interestingly, in the computer condition the opposite direction was found. Participants who estimated their knowledge as high tended to choose the high prize lottery less excessively than those who estimated their knowledge as low. This finding seems to correspond with our finding in Experiment 1, where participants who felt highly skillful tended to under estimate their chances to enter the lottery in the computer condition. As noted above, this suggests that feeling skillful does not necessarily lead to general over-confidence or optimism.

Noticeably, the collective behavior in the self and in the computer conditions reflects close-to-perfect coordination. This may be due to the variance in participants’ knowledge estimates. Namely, in the self condition, participants who estimated their skill as high over-chose the high-prize lottery, and those who estimated their skill as low under-chose the high-prize lottery, and vice versa in the computer condition. Consequently, in both conditions we found coordination overall.

The idea that individual differences may account for the coordination observed was recently suggested by Bereby-Meyer et al. (in press). In their study, participants were more likely to choose the low-prize lottery, the higher their analytical thinking style and the more risk-averse preferences they expressed. The authors suggest that variance in participants’ risk tendency and in thinking style accounts for the observed coordination [2].

In line with this notion, the findings we report here suggest that variance in self-skill perception may explain the high levels of coordination observed in previous studies. Specifically when variance in self-skill perception is considerable, some decision makers feel highly skillful and thus overly choose one option whereas others feel unskilled and thus overly choose the other option, leading to coordination at the overall group level (for a more detailed explanation of how variance may lead to coordination see [2]). Contrarily, in domains and situations in which skill is homogeneously perceived as high (i.e. with low variance), coordination will be difficult to achieve. Most people are likely to focus on their high skills and fail to consider others’ decisions when making their choice.

Results of this research may shed light on phenomena in the real world where feelings of skillfulness are likely to play a role. For example, the coordination failure often observed in the area of entrepreneurial entry decisions may be accounted for by skill overconfidence [17], [18], [19]. Indeed, Koellinger et al. (2007) found entrepreneurs to be more confident in their skills and abilities than non-entrepreneurs, and this confidence was negatively correlated with survival rates of nascent entrepreneurs. Also, Cooper et al. (1988) found that entrepreneurs were overly optimistic about their prospects, much like our participants, who were overconfident about their chances to enter the lottery, when their self skill appeared to be related to the outcome.

## Supporting Information

Text S1 Instructions for Experiment 1 for the computer condition.
(DOCX)

Text S2 Instructions for Experiment 2 for the computer condition.
(DOCX)
