# Supplementary material for: When Feeling Skillful Impairs Coordination in a Lottery Selection Task
Source: PLoS One. 2013 Jun 14;8(6):e65092. doi: 10.1371/journal.pone.0065092 (PMC3683036; doi:10.1371/journal.pone.0065092)
Supplement: Text S1 — Instructions for Experiment 1 for the computer condition. (DOCX) [file pone.0065092.s001.docx]

Text_S1: Instructions for Experiment 1 for the computer condition.

You will now have the chance to participate in one out of two cash-prize lotteries. Forty participants (including you) with similar high scores will participate in these lotteries.

You may choose one of the following lotteries:

(1) "Roni Game"

Phase 1: One trial of the IQP task.

This trial is different from the task you just performed! Instead of you estimating how many dots compose the target, a computer program will estimate the number of dots for you. The chance of the computer program making a correct estimation is close to 99%. The estimation made by the software will serve as your estimation of the number of dots.^[[1]](#footnote-1)^

Phase 2: Of all participants with a correct estimation, one winner will be randomly selected to receive a prize of 100 IS.

(2) "Adi Game"

Phase 1: One trial of the IQP task.

This trial is different from the task you just performed! Instead of you estimating how many dots compose the target, a computer program will estimate the number of dots for you. The chance of the software making a correct estimation is close to 99%. The estimation made by the program will serve as your estimation of the number of dots.

Phase 2: Of all participants with a correct estimation, one winner will be randomly selected to receive a prize of 200 IS.

Please note: Phase 1 is identical in both games.

1. Which game do you choose to enter – the "Roni Game" or the "Adi Game"?

Please answer the following questions:

1. Estimate the number of participants (out of 40) who will choose the "Roni Game".
2. Estimate the number of participants (out of 40) who will choose the "Adi Game".
3. Estimate your IQP skill on a scale of 1-9 (1 means very low skill and 9 means very high skill).
4. Estimate the IQP skill of another participant (out of the 40 participants), on a scale of 1-9 (1 means very low skill and 9 means very high skill)
5. Estimate the chance that the program will choose the correct digit for you, on a scale of 1-9 (1 means no chance and 9 means certainly).
6. Estimate the chance that the program will choose the correct digit for another participant (out of the 40 participants), on a scale of 1-9 (1 means no chance and 9 means certainly).
7. Estimate the difficulty level of the IQP task you performed, on a scale of 1-9 (1 means a very easy task and 9 means a very difficult task).
8. Please indicate how the next trial of the IQP task will be performed:

a. I will estimate the number of dots presented.

b. A computer program will choose a digit that will serve as my estimation.

1. In the self condition participants received identical instructions. The only difference was that participants were told that in both the "Adi" and "Roni" games they must estimate correctly how many dots compose the target by pressing the correct digit. They also evaluated their and another participant's chance of making a correct estimation (questions 6-7). [↑](#footnote-ref-1)
