# Supplementary material for: When Feeling Skillful Impairs Coordination in a Lottery Selection Task
Source: PLoS One. 2013 Jun 14;8(6):e65092. doi: 10.1371/journal.pone.0065092 (PMC3683036; doi:10.1371/journal.pone.0065092)
Supplement: Text S2 — Instructions for Experiment 2 for the computer condition. (DOCX) [file pone.0065092.s002.docx]

Text_S2: Instructions for Experiment 2 for the computer condition

Dear student,

Imagine that 200 participants, including you, are given the opportunity to participate in one out of the two following games:

(1) "Roni Game"

In this game you must correctly guess the outcomes of three upcoming soccer games

Maccabi Tel Aviv vs. Hapoel Haifa

Bnei Sahnin vs. M. S. Ashdod

Hapoel Beer Sheva vs. Maccabi Haifa

In this game, a random device will guess the outcomes of the games for you. You will not have any influence on this guess.^[[1]](#footnote-1)^ If the guesses made for you are correct you will win 250 IS. If the guesses made for more than one participant are correct, one of these participants will be randomly selected to win 250 IS.

(2) "Adi Game"

In this game you must guess correctly the outcomes of three upcoming soccer games

Maccabi Tel Aviv vs. Hapoel Haifa

Bnei Sahnin vs. M. S. Ashdod

Hapoel Beer Sheva vs. Maccabi Haifa

In this game, a random device will guess the outcomes of the games for you.

You will not have any influence on this guess. If the guesses made for you are correct you will win 500 IS. If the guesses made for more than one participant are correct, one of these participants will be randomly selected to win 500 IS.

1. Which game do you choose to enter – the "Roni Game" or the "Adi Game"?
2. Estimate the number of other participants (out of 200) who will choose the "Roni Game".
3. Estimate the number of other participants (out of 200) who will choose the "Adi Game".
4. Rate your knowledge in soccer on a scale of 0-10 (0 means no knowledge at all and 10 means perfect knowledge).

1. In the skill-relevant condition participants received identical instructions. The only difference was that participants were told that they must correctly guess the outcomes of three upcoming soccer games in both games. [↑](#footnote-ref-1)
